# The challenge of radiology education in developing countries

**DOI:** 10.2349/biij.4.1.e2

**Published:** 2008-01-01

**Authors:** JL Ramírez-Arias, C Rodríguez-Treviño, C González-Vergara

**Affiliations:** 1 School of Medicine, Universidad Nacional Autónoma de México, Mexico; 2 School of Medicine, Universidad La Salle México, Mexico

## INTRODUCTION

Radiology education has been carefully analysed in WHO radiological education meetings [1]. Among several important issues, the economical influence was discussed. The percentage of gross national product (GNP) in health is important in countries where it has not been progressively increased. This is the reality in many developing countries with low and medium income. It has a bad impact on their health system budgets [2]. This condition makes it impossible to have modern infrastructure and usually, there is a lack of well-trained personnel and well-trained staff. Most hospital certifications, if any, are impossible to obtain and academic stimulus for academic staff is usually scarce. These points have influenced radiology education at a time where new radiologists must be prepared for all the modern imaging modalities and also for the scientific advances including molecular imaging, genomic medicine, and the use of high technology, such as radiology computed equipment including Picture Archiving Communication Systems (PACS). Public and university hospitals have traditionally been related to the training of medical residents - they have large clinical grounds and their patients are less inconvenienced when they receive medical treatment by the institution’s personnel as opposed to private institutions where the physicians are usually chosen by the paying patient.

Things are changing as academic radiologists, usually part of the public or university hospitals’ staff, are emigrating to private practices where remunerations are much better and the infrastructure is frequently more sophisticated and updated. The new concept of concierge medicine [3] allows physicians to avoid attending to a massive number of patients. Therefore, they tend to have a more relaxed medical practice but most of the time away from scholastic purposes. Unfortunately, less experienced radiologists remain in the academic institutions and they are usually in charge of postgraduate radiology education.

The radiology programs can become obsolete, impeding the training of adequate new radiologists who frequently failed their last exams or certification board tests, in countries where this requisite exists.

Academic radiologists have little stimulus and this must be modified. It is hardly fair that they have to finance educational material which can be very expensive and of course research can be even more difficult to perform.

Low health budgets favour stillness and, most frequently, obsolete standards and regulations, which impede modernisation in medical practice, including high-quality radiology standards that could be conducive to the enrichment of radiology education [4].

High-technology equipment is part of the requirements of modern radiology; helical CT scanners, less than 1.0 tesla MRIs and ultrasound without Doppler capabilities are considered obsolete, impeding again adequate and modern radiology education.

Private medical institutions supported by private insurance companies and health maintenance organisations (HMOs), expedite administrators in the knowledge that the relation of cost-benefit while investing in costly radiologic equipment will usually give them the benefit of a faster capital reimbursement if they have competitive equipment to offer.

Some large private hospitals are now interested in promoting education, among other reasons because they have manpower requirements and residents can be part of low-cost medical staff while in training, they also know that academic personnel as part of the staff improves the prestige of the institution.

If this is the future, let’s do it right. If we have high-standard radiologists and high-technology equipment, the bases are settled to motivate radiologists to participate and contribute in the formation of new high-quality radiologists [5]. The association with postgraduate university programs is essential, appointment of staff radiologists by a university institution is a good motivation to participate and in addition, they can have additional income, which is usually small.

PACS are now a reality, again most are set up in private hospitals. In addition to the clinical advantages of this costly administration system equipment, the educational value must be considered. Modern radiology education is based on the new concepts of adult learning [6] and evidence-based on radiology programs [7].

It is important that radiologists qualify in teaching, they will later have the responsibility of training residents to be professionals of high quality and standard.

Learning must be the main motivation for anyone in training and it should be based on what he or she wants to learn, and not what the mentor can or wants to teach. Here is where electronic education plays an essential role for the radiology residents: they can have access to large databases and many radiology education files.

With these tools, cognostic issues can be improved but the need for instructors and mentors, now called facilitators, will always exist. Furthermore, if a good technological infrastructure is available it will support diagnostic and interventional procedures thus improving the trainees’ skills and stimulating residents.

Professional medical colleges aim to have duties and medical privileges for their members based on good medical practice. The support of radiological colleges will help in many ways to modernize and update standards that guarantee a high quality radiology performance in places, which lack standards and regulations.

Radiologists with professional recognition from their peers with ample experience must try to support the development of this kind of institutions including the creation of certification and bioethics bodies. With these achievements, radiology education can be progressively and continuously developed to the highest standards possible.
